# Activation of atomically precise silver clusters on carbon supports for styrene oxidation reactions†

**DOI:** 10.1039/c9ra05566e

**Published:** 2019-09-06

**Authors:** Kazeem O. Sulaiman, V. Sudheeshkumar, Robert W. J. Scott

**Affiliations:** Department of Chemistry, University of Saskatchewan 110 Science Place Saskatoon Saskatchewan S7N 5C9 Canada robert.scott@usask.ca

## Abstract

Metal clusters have distinct features such as large surface area, low-coordination-atom enriched surfaces, and discrete energy levels that influence their behavior during catalytic reactions. Atomically-precise Ag clusters, which are analogues of more well-studied Au clusters, are yet to be fully explored as catalysts for various chemical reactions. 2,4-Dimethylbenzenethiol-protected Ag_25_ clusters were prepared and deposited onto carbon supports followed by calcination. Results from X-ray absorption fine structure (EXAFS) spectroscopy measurements and other characterization techniques indicated that thermal activation of carbon-supported Ag_25_ clusters resulted in dethiolation of Ag clusters at 250 °C and beyond, and consequently mild growth in particle sizes of Ag clusters on carbon supports was seen with increasing activation temperatures. Both as-prepared and activated Ag_25_ clusters were active for styrene oxidation reactions, with high selectivity towards styrene oxide, without using any promoter. Results show that mild activation at 250 °C yields the most active catalysts, and higher activation temperatures lead to decreased activities and slightly poorer selectivity to styrene oxidation as a result of cluster sintering. EXAFS data shows the resulting activated clusters are composed of Ag metal and that all thiols are removed from the Ag cluster surfaces, though XPS data shows that thiol oxidation products are still present in the sample.

## Abstract 2

Atomically-precise silver clusters can be activated for styrene oxidation reactions using mild calcination conditions.

## Graphical abstract

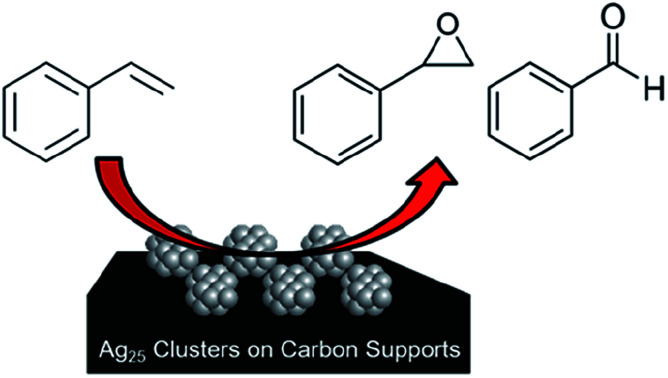

## Introduction

The use of well-defined clusters for the development of model heterogeneous catalysis is a very active research area.^1,2^ Noble metals with sizes in the nanoscale generally show excellent catalytic activity due to their enhanced surface-to-volume ratio which leads to more active sites, as well as having modified surface geometric and electronic properties compared to bulk materials.^3,4^ By tailoring the size as well as the morphology and composition, robust nanocatalysts with excellent catalytic activity and selectivity can be achieved.^4^ Atomically-precise thiolate ligand protected clusters are widely studied due to strong sulfur–metal interactions that enable good stability in solution, facile synthesis, and controlled cluster compositions as well as functionalization of stable clusters.^5^ Owing to possible influence of the capping ligand on the activity and/or selectivity of ligand-protected clusters in catalytic reactions, it is desirable to have partial or complete ligand removal to enhance contact between the surface metal atoms and reactants, and thus allow higher catalytic activity.^5,6^ This leads to a research challenge in preventing or controlling particle aggregation upon removal of ligands from the protected clusters at high loading on supports. Metal-support interactions can play a key role in stabilizing naked clusters, in addition to other strategies that involve creating physical barriers to minimize or prevent particle aggregation.^7,8^

Many research groups, including ours, have extensively studied Au_25_(SR)_18_^−^ clusters for various catalytic reactions.^9–15^ X-ray absorption near edge structure (XANES) and extended X-ray absorption fine structure (EXAFS) techniques are components of X-ray absorption spectroscopy (XAS) which are often used in combination to probe chemical bonding and local structure properties of metal clusters with thiol ligands.^12,16,17^ Many research groups have demonstrated that XAS is a reliable bulk technique for following particle size growth upon thermal activation of supported Au clusters.^12,18–20^ Jin and coworkers showed that Au–thiolate bonds are stable to heating until 125 °C beyond which the metal clusters become deprotected.^21^ A number of research groups have observed sintering of Au clusters upon thermal activation at temperatures above 250 °C for supported clusters, though some supports show strong interaction with clusters which mitigate sintering.^10,12,22–25^ Other activation strategies include chemical approaches, which involve the use of oxidizing and reducing agents, as well as light-induced approaches.^26–30^ Both naked and protected Au_25_ clusters have been reported to be active catalysts for styrene oxidation,^10,31^ and several groups including our group have shown size-dependent activity of Au clusters, with high selectivity towards styrene oxide.^10,13^ Ag nanoparticles have also shown to be effective catalysts for styrene epoxidation, though the use of a secondary promoter is a common practice to achieve high selectivity of the epoxide product.^32,33^ Then, selective styrene epoxidation can serve as a good model oxidation reaction to assess the activity of atomically-precise Ag cluster catalysts.

A large number of ligand-protected Ag clusters such as Ag_9_, Ag_10_, Ag_16_, Ag_25_, Ag_29_, Ag_32_ and Ag_44_ have been reported in the literature.^20,34–38^ Ag_25_(SR)_18_^−^ clusters have similar (but not identical) atomic arrangements and ligand counts as Au_25_(SR)_18_^−^ clusters. Bakr and coworkers reported the successful synthesis and structural elucidation of Ag_25_(SR)_18_^−^ clusters and noted that the crystal structure has four voids that can allow solvent coordination to give better stability of the clusters.^5,39^ The predisposition of silver (Ag) to oxidation, in spite of its lower cost and higher relative abundance (compared to Au), imposes possible limitations on the development of heterogeneous catalysts based on thiolate-protected Ag clusters. Indeed, early work in the field suggested that Ag systems might behave significantly differently than analogous Au systems. Padmos and Zhang showed by XAS that as-synthesized small thiolate protected Ag nanoparticles look to have Ag cores and Ag_2_S shells, while dialkylsulfide-stabilized Ag nanoparticles have pure Ag cores.^40^ Pradeep and coworkers found that glutathione-stabilized Ag_25_L_18_ clusters formed Ag_2_S materials, as evidenced by PXRD, after heating at 80 °C in solution.^36^ However, later work by Tsukuda and coworkers employed XAS techniques to study the behavior of mesoporous carbon supported [Ag_44_(SC_6_H_4_F)_30_]^−^ clusters upon thermal treatment.^20^ They observed sulfur-free Ag clusters upon calcination at 300 °C, which were used as catalysts for the catalytic dehydrogenation of ammonia-borane. At the current time, it is not clear whether such discrepancies in results are due to ligand differences or due to improved purification protocols of Ag cluster materials. This manuscript employs XAS and other techniques to probe the behavior of 2,4-dimethylbenzenethiolate-protected Ag_25_ clusters upon thermal activation for heterogeneous catalysis.

In this study, the structural and morphological properties of the purified Ag_25_L_18_^−^ clusters, as catalyst precursors, were evaluated using characterization techniques such as UV-vis spectroscopy, thermal gravimetric analysis (TGA), and transmission electron microscopy (TEM), followed by monitoring the effect of choice of coordinating solvents and storage temperature on the stability of solvent dispersed Ag_25_L_18_^−^ clusters. Thereafter, Ag_25_L_18_^−^ clusters were supported on carbon supports and activated thermally, followed by characterization of carbon-supported Ag_25_ clusters using X-ray photoelectron spectroscopy (XPS), TEM, and XAS, and then examined for catalytic activity and selectivity for styrene oxidation reactions. Our results show that Ag–thiol bonds are selectively oxidized from the clusters upon mild heat treatments without formation of Ag_2_O or Ag_2_S, and that the activated Ag clusters on carbon supports showed particle size-dependent activity for styrene oxidation reactions.

## Experimental

### Materials

All chemicals are commercially available and used as received without any further purification. Silver nitrate (AgNO_3_, ≥99.0%), styrene (ReagentPlus®, 99.9%), *tert*-butyl hydroperoxide (*t*BuOOH, TBHP, 70% in H_2_O), high purity acetonitrile (MeCN) and 100% ethanol (EtOH) were purchased from Sigma Aldrich. Dichloromethane (CH_2_Cl_2_, DCM), methanol (MeOH, HPLC grade), sodium borohydride (NaBH_4_, 98%), tetrahydrofuran (high purity, THF) were purchased from Fisher Scientific. 2,4-Dimethylbenzenethiol (HSPhMe_2_, C_8_H_9_SH, 95%) and tetraphenylphosphonium bromide (Ph_4_PBr) were purchased from Alfa Aesar. Dimethylformamide (DMF, HPLC grade), dimethylsulfoxide (DMSO, HPLC grade), and activated carbon (CX0657-1) were purchased from EMD. Milli-Q (Millipore, Bedford, MA) deionized water (resistivity 18.2 MΩ cm) was used in all experiments.

### Synthesis and purification of the alkanethiolate-protected Ag_25_ clusters

The Ag_25_L_18_^−^ (where L is SPhMe_2_) clusters were synthesized using a literature procedure,^39^ with slight modifications. In a typical synthesis, 2.0 mL of MeOH was used to dissolve 0.22 mmol of AgNO_3_ in 50 mL flask with 5 min of sonication. Addition of 0.66 mmol of 2,4-dimethylbenzenethiol to this solution gave a thick yellow mixture that became dispersed with the addition of 18 mL of DCM. This solution was kept in an ice-bath with constant stirring at 600 rpm for 30 min before adding a freshly prepared 0.50 mL methanolic solution containing 0.014 mmol of PPh_4_Br. Afterwards, 0.50 mL of an ice-cold aqueous solution containing 0.40 mmol of NaBH_4_ was added dropwise and the reaction mixture was continuously stirred in an ice-bath for 6 h. Within this period, the colour of the solution changed from deep yellow to light yellow and finally to a dark red, which indicates the reduction of the silver(i)-thiolate mixture.

The crude solution was aged at ∼4 °C for 18 h followed by centrifugation (8500 rpm for 30 min) to remove a solid that presumably constitutes the excess thiol ligands. The obtained supernatant was then concentrated under rotary evaporator to about 5 mL before adding 20 mL of methanol to precipitate the Ag clusters. The precipitate was collected by centrifugation and extracted into 20 mL of DCM. The DCM solution of the clusters was again centrifuged (8500 rpm for 60 min) to remove an insoluble yellow solid. Then, the deep red colored supernatant was dried under vacuum, and the obtained purified and dried [Ph_4_P][Ag_25_L_18_] clusters were stored in a glass vial under ambient conditions for further studies.

### Preparation of carbon-supported Ag_25_L_18_^−^ clusters

Immobilization of Ag_25_L_18_^−^ clusters onto carbon supports was done *via* the wetness impregnation method, to give a final metal loading of *ca.* 4 wt%. Typically, *ca.* 15.5 mg of Ag_25_ clusters was dissolved in 5.0 mL of DCM followed by adding 200 mg of activated carbon and stirring for 24 h. Afterwards, the carbon-supported Ag_25_L_18_^−^ clusters (Ag_25_/carbon) were first dried at 110 °C for 2 h in an oven, and then thermally treated at 250 °C, 350 °C, or 450 °C, for 2 h, at a ramping rate of 10 °C min^−1^ under air flow using a Lindberg/Blue M furnace. The as-prepared and thermally-treated Ag_25_/carbon samples were analyzed with TEM, XPS, and XAS (see below).

### Characterization techniques

UV-vis absorption spectroscopy spectra of the Ag_25_L_18_^−^ clusters in solution were obtained using a Varian Cary 50 UV-visible spectrophotometer with an optical path length of 1 cm. A very small amount of the dried Ag_25_L_18_^−^ clusters was dissolved in 2.0 mL of suitable solvent (*e.g.* DCM) to prepare a sample solution for UV-vis measurements. Thermal gravimetric analyses were performed using a TA Instruments TGA Q5000IR, upon which samples were heated from 25 to 800 °C under an air flow, with a heating rate of 5 °C min^−1^.

Transmission electron microscopy (TEM) images were collected using a Hitachi HT7700 and HF 3300 TEM operated at 100 kV and 300 kV, respectively. TEM samples were prepared by drop casting the Ag_25_L_18_^−^ cluster solution onto a carbon coated 300 mesh copper TEM grid (Electron Microscopy Sciences, Hatfield, PA) and dried at ambient condition prior to TEM analysis. In the cases of thermally activated Ag_25_ clusters on carbon, each sample was dispersed in EtOH, and the dispersion was dropped onto a carbon coated copper grid, followed by drying the sample grid at ambient conditions for 2 h prior to TEM analysis. Average particle sizes were typically determined by manually measuring 100 particles from images obtained for each sample using the ImageJ software program.^41^

X-ray photoelectron spectroscopy (XPS) measurements were collected using a Kratos (Manchester, UK) AXIS Supra system at the Saskatchewan Structural Sciences Centre (SSSC). This system is equipped with a 500 mm Rowland circle monochromated Al Kα (1486.6 eV) source and combined hemi-spherical analyzer (HSA) and spherical mirror analyzer (SMA). An accelerating voltage of 15 keV, an emission current of 15 mA, and a hybrid spot size of 300 × 700 microns were used. All survey scan spectra were collected in the −5 to 1200 eV binding energy range in 1 eV steps with a pass energy of 160 eV. High resolution scans of three regions were also conducted using 0.05 eV steps with a pass energy of 20 eV. All binding energies were calibrated to the C 1s of the adventitious carbon at 284.8 eV and spectra were fitted without constraining the full width at half maximum (FWHM) using CasaXPS software.^42^

The Ag K-edge XAS measurements were conducted at the Hard X-ray MicroAnalysis (HXMA) beamline 061D-1 (5–30 keV; resolution 1 × 10^−4^ Δ*E*/*E*) at the Canadian Light Source (CLS). Energy selection of the Ag K-edge was done using a Si(220) double-crystal Si monochromator, with a Pt-coated water-cooled collimating KB mirror. Ionization chambers were filled with nitrogen gas. Samples were mixed with boron nitride and measured in fluorescence mode at room temperature using a 32-element detector. The energy of the X-rays was calibrated by using Ag foil. The data processing was performed using the IFEFFIT software package.^43^ The X-ray absorption near-edge structure (XANES) spectra were obtained by subtracting the atomic absorption background and normalizing the spectra to the edge height. The *k*^3^-weighted spectra were subjected to a Fourier transform (FT) in *R* space for the *k* range of 2.8–12 Å^−1^. Ag fcc bulk lattice parameters were used to fit the Ag-foil spectrum, keeping the first shell coordination number fixed (CN = 12) at first, in order to determine the amplitude reduction factor (S_0_^2^). From this fitting, the amplitude reduction factor determined for the Ag-foil was 0.80 and was used for subsequent sample fits.

### Catalytic measurements for styrene oxidation reactions

The catalytic performances of the as-prepared and activated Ag_25_/carbon samples were evaluated for styrene epoxidation reactions. For each catalyst sample, the catalytic test was conducted inside a 100 mL round bottom flask fitted with a reflux condenser. A typical reaction set-up includes addition of styrene (920 μL, 8.0 mmol), TBHP (3.3 mL, 24 mmol), MeCN (5.0 mL), and catalyst (20 mg) into a flask under reflux and stirring (600 rpm) at a reaction temperature of 82 °C for 24 h.^13^ Products were then analyzed using a gas chromatograph (7890A, Agilent Technologies) equipped with a HP-5 column and a flame ionization detector.

## Results and discussion

**Fig. 1:**
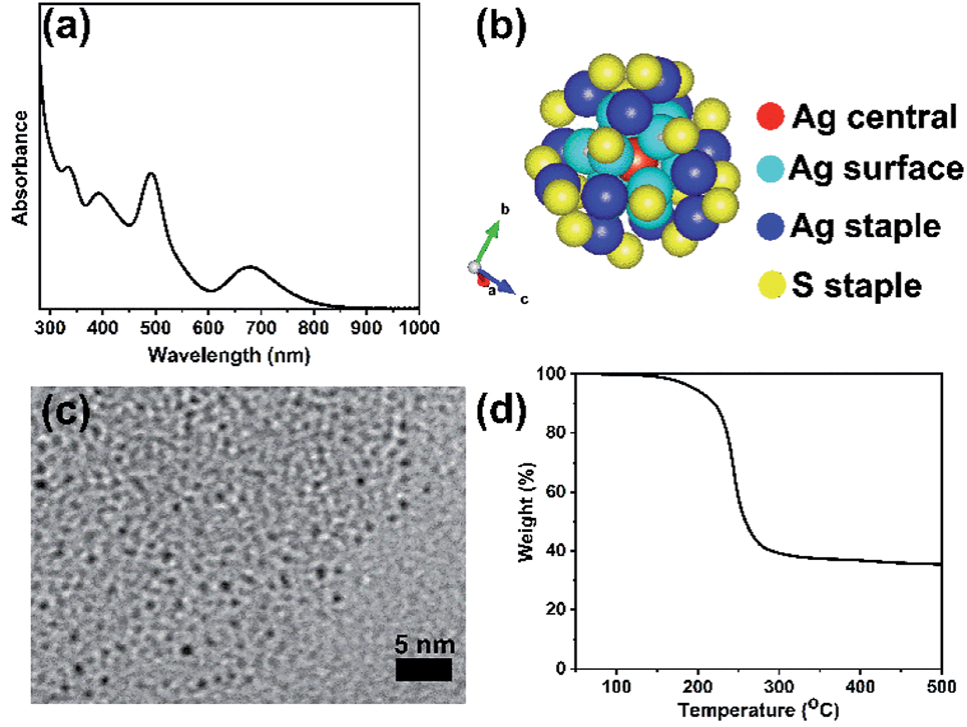
Characteristic features of Ag_25_L_18_^−^ clusters (a) UV-vis spectra in DCM, (b) atomic arrangement as visualized using VESTA software,^44^ colour scheme is red for the Ag central atom, light blue for Ag surface atoms, dark blue for Ag staple atoms and yellow for sulfur staple atoms. (c) TEM image, and (d) TGA plot.

The formation of a deep-red solution upon addition of sodium borohydride to the Ag(i)-thiolate complex indicates the reduction of Ag(i) ions and formation of thiolate protected silver clusters. Fig. 1 presents characterization results obtained for purified, unsupported thiolate-protected metal clusters consisting of precisely 25 silver atoms; Ag_25_L_18_^−^, where L is –SPhMe_2_. Fig. 1(a) shows the UV-vis spectrum of purified Ag_25_L_18_^−^ clusters in DCM with characteristic peaks at 334 nm, 392 nm, 490 nm and 678 nm for nearly monodisperse Ag_25_L_18_^−^ clusters.^39^ Similar to the case for Au_25_ clusters, the broad peak at around 678 nm is due to the HOMO–LUMO transition due to the Ag_13_ icosahedral core in the Ag_25_ structure. Fig. 1(b) shows the previously determined crystal structure of Ag_25_L_18_^−^,^39^ as visualized using the VESTA software package,^44^ to reflect the atomic arrangement and ligand counts. There is one silver atom (red) at the center and twelve silver atoms (dark blue) on the surface of the icosahedral core. The remaining twelve silver atoms (blue) and eighteen sulfur atoms (yellow), from thiolate ligands, constitute atoms in the staple positions. Fig. 1(c) shows a TEM image which shows individual Ag_25_L_18_^−^ clusters have an average size of 1.0 ± 0.2 nm. This measurement slightly lesser than a value of 1.5 ± 0.3 nm previously reported for Ag_25_(SR)_18_,^45^ but closer in line to the expected size of the Ag core. The slight difference in measured average particle size can be due to better size separation, due to extra purification step, in our synthesis procedure, as well as moderate voltage imaging at 100 kV on new sample areas to minimize damage to the clusters by the electron beam. The TGA result in Fig. 1(d) shows no weight loss below 150 °C and a major weight loss centering around 250 °C, which is presumed to be due to removal of the protecting thiolate ligands from the Ag_25_L_18_^−^ clusters upon thermal treatment. The final weight percent of material leftover is approximately 40%, which is slightly lower than the theoretically estimated *ca.* 47% Ag composition of the clusters and counter ions, *i.e.* Ph_4_P[Ag_25_(SR)_18_].

Table S1 (ESI†) summarizes the preservation of UV-vis spectroscopy fingerprints of Ag_25_L_18_^−^ clusters in four different aprotic polar solvents (THF, DMF, DCM, and DMSO), under different storage conditions. Equal concentrations (0.10 mg mL^−1^) of Ag clusters were prepared with different solvents and time-dependent UV-vis studies were conducted for clusters stored at room (22–24 °C) and cold (∼4 °C) temperatures. The fingerprints of Ag_25_L_18_^−^ clusters in the UV-vis spectroscopy measurements turn out to be good features for evaluating the stability of clusters in these solvents. The voids in the crystal structure of Ag_25_L_18_^−^ allow coordination with suitable solvents, and thus can be correlated with the solution-based stability of the Ag_25_L_18_^−^ clusters.^5,39^ The values of dielectric constant of the selected solvents correlates with the number of days it takes for the precipitation of the Ag_25_L_18_^−^ clusters, presumably due to loss of coordination of solvent molecules with the voids in the crystal structure of Ag_25_L_18_^−^ clusters. Both the nature of coordinating solvent and storage temperature affect the solution-based stability of Ag_25_L_18_^−^ clusters. Importantly, it takes a minimum of 48 h to observe precipitation of Ag_25_L_18_^−^ clusters in any of these solvents at room temperature and thus any choice among these solvents is suitable for immobilization of Ag_25_L_18_^−^ clusters onto carbon supports *via* wetness impregnation, a procedure that was completed under 24 h, in making heterogeneous catalysts. DCM solvent was chosen for immobilization procedure as it dries quickly due to its lower boiling point.

**Fig. 2:**
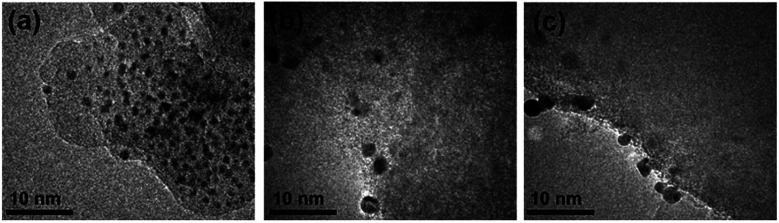
TEM images of thermally activated Ag_25_/carbon catalyst samples at (a) 250 °C (b) 350 °C, and (c) 450 °C.

The activation of heterogeneous catalysts derived from colloidal precursors often entails partial or complete removal of any protecting ligands to enable access of active sites by reactants^21,46–48^ While the activation can be achieved by several approaches, thermal calcination approaches to activation are quite simple and thus employed in this present study. TEM, XPS, and XAS analyses of the Ag_25_/carbon samples gave insight into the extent of ligand removal and particle growth at different activation temperatures. Fig. 2 shows TEM images of thermally-activated Ag_25_/carbon catalysts. It is noteworthy that imaging clusters on carbon supports was somewhat difficult due to the small size and poor contrast between the support and clusters. Nevertheless, no appreciable increase in cluster size was noted upon loading the Ag clusters onto carbon supports. This suggests that majority of Ag clusters are effectively adsorbed without structural compromise. Improved contrast was seen between Ag and the carbon support for Ag_25_/carbon samples calcined at 250 °C and beyond. Average particle sizes were 1.2 ± 0.3 nm, 2.6 ± 0.4 nm, and 2.8 ± 0.6 nm for activated samples at 250 °C, 350 °C, and 450 °C, respectively. This shows that very minor growth in the average cluster size is seen for samples calcined at 250 °C, while the cluster size grows progressively with higher activation temperatures. Thus, particle growth revealed by TEM images may be a consequence of the removal of protecting thiolate ligands upon thermal activation of as-prepared Ag_25_/carbon catalysts.

**Fig. 3:**
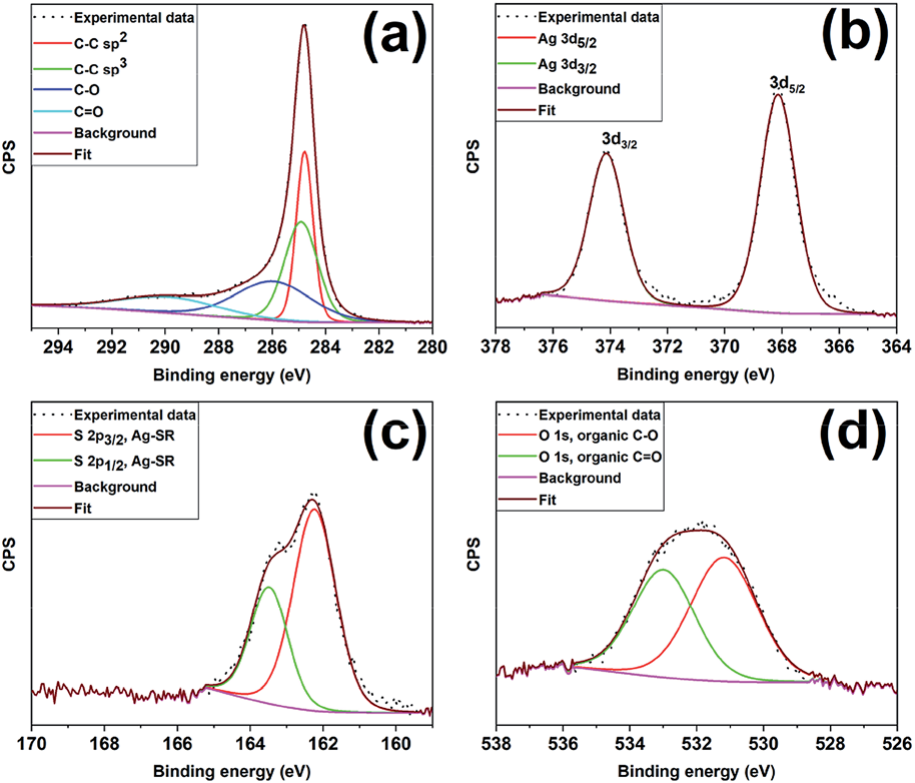
XPS spectra for the as-prepared Ag_25_/carbon catalyst; (a) C 1s (b) Ag 3d, (c) S 2p and (d) O 1s peaks.

Fig. 3 shows the XPS spectra of the constituent elements of the as-prepared Ag_25_/carbon sample; XPS survey maps show the presence of C, Ag, S, and O as the constituent elements. The C 1s spectrum was fitted with multiple Gaussian components having peaks with binding energies at 284.8 eV, 284.9 eV, 285.9 eV, and 289.9 eV and these can be attributed to C–C sp^2^, C–C sp^3^, C–O, and C=O species respectively; the latter species are commonly found on activated carbon surfaces.^49^Fig. 3(b) shows the Ag 3d_5/2_ and Ag 3d_3/2_ peaks at 368.1 eV and 374.1 eV, respectively, which are consistent with silver atoms in the zerovalent state (Ag 3d_5/2_ = ∼368.0 eV).^50,51^ Deconvolution of the high resolution spectrum of S 2p spectrum, shown in Fig. 3(c), shows two peaks with spin–orbit splitting of ∼1.2 eV indicating the presence of Ag-thiolate species (162.2 eV for S 2p_3/2_ and 163.5 eV for S 2p_1/2_).^52^ The O 1s spectrum shows an asymmetric profile that suggests the presence of more than one kind of oxygen species (Fig. 3(d)) on the carbon surface. The deconvolution of the spectrum gives two peaks centering at ∼531.2 and 533.1 eV that can be ascribed to C–O and C=O species from the activated carbon surface, respectively, and thus the absence of metal oxide species.^53^ Altogether, XPS results indicate that thiolate ligands remain attached to the surface of silver cluster surface and no oxidation of silver atoms occurs in the Ag_25_/carbon sample prepared *via* wetness impregnation.

**Fig. 4:**
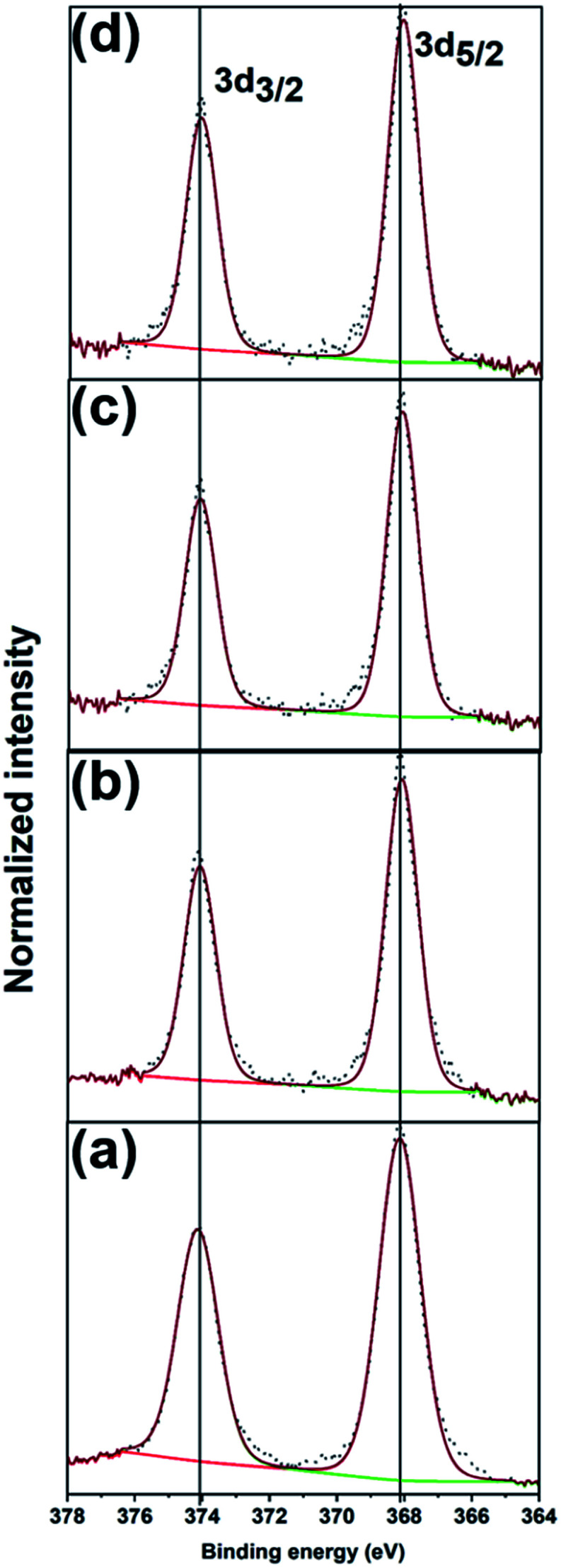
XPS spectra of Ag 3d peaks for (a) as-prepared, and the calcined Ag_25_/carbon catalyst samples at (b) 250 °C, (c) 350 °C, and (d) 450 °C.

Fig. 4 shows the XPS spectra and fits of the Ag 3d peaks for the calcined Ag_25_/carbon catalyst samples. In the calcined samples, the Ag 3d_5/2_ and Ag 3d_3/2_ peaks are at 368.03 eV and 374.03 eV respectively, which show that silver atoms maintain the zerovalent state after calcination. In contrast, analysis of the S 2p spectra of activated samples shows the absence of peaks earlier ascribed to Ag-thiolate species (Fig. S1, ESI†). Instead, S 2p peaks appears at higher binding energies and these peaks can be attributed to the organic disulfide and other oxidized sulfur species like sulfonates,^54,55^ which are byproducts of oxidative dethiolation. Also, there is no indication of formation of silver oxide or sulfide from both Ag 3d and O 1s spectra obtained for activated sample (Fig. 4 and S1, ESI†). Also, from the quantification of survey spectra, the amount of sulfur species decreases with increase in activation temperatures. The sulfur species content was quantified to be 1.40% in the as-prepared sample, 0.55%, 0.14% and ∼0.01% for activated samples at 250 °C, 350 °C, and 450 °C respectively. These XPS results suggest that thiolate ligands leave the surface of silver clusters upon thermal activation, which is consistent with the TGA results (Fig. 1(d)). Zhang *et al.* recently reported a similar observation showing migration of protecting ligands from the gold metal surface onto an oxide support upon deposition and oxidative pretreatment.^56^ All XPS results suggest Ag clusters remain in the zerovalent state after calcination, and that no significant Ag oxidation is occurring upon calcination.

**Fig. 5:**
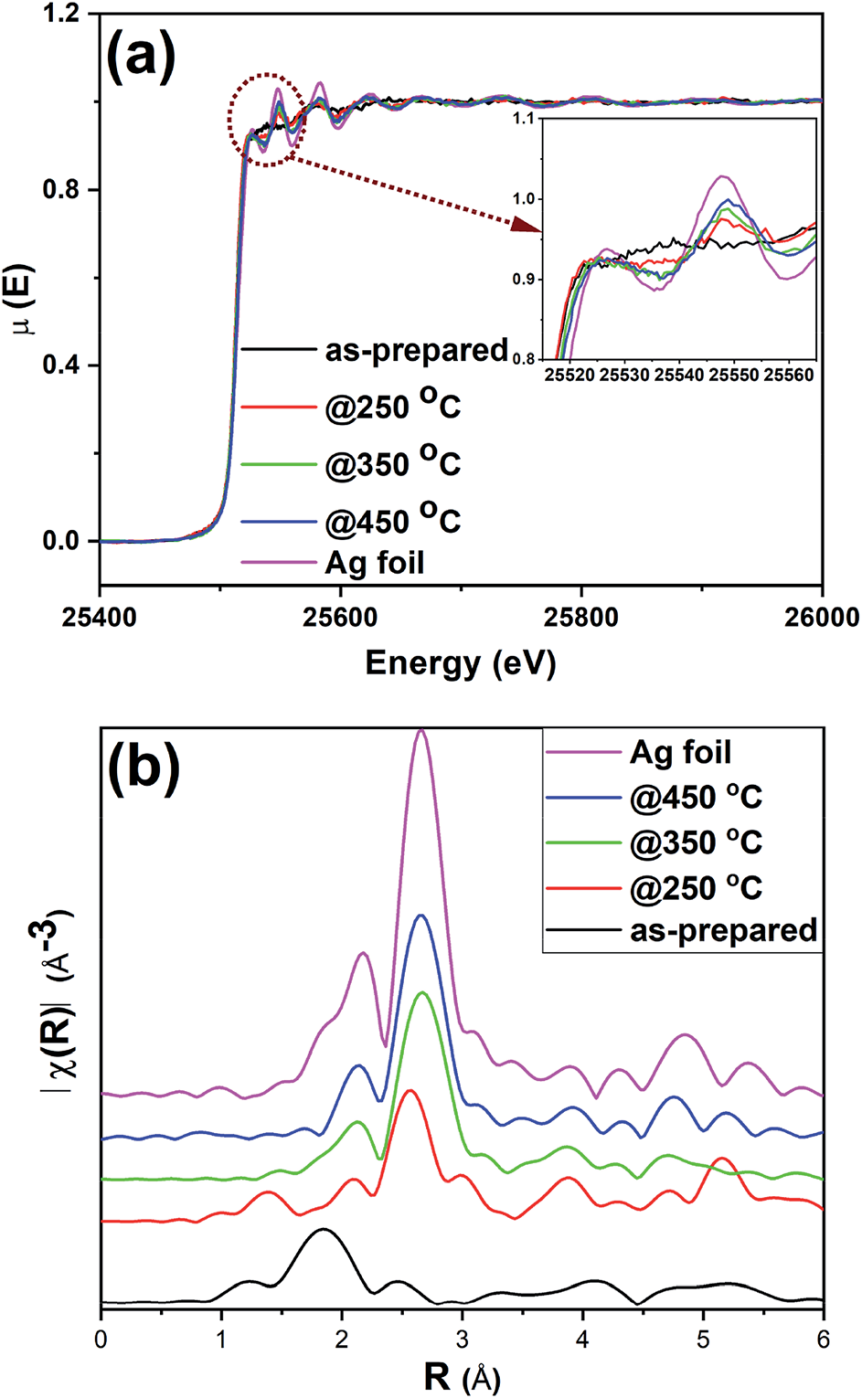
Ag K-edge (a) XANES and (b) FT-EXAFS spectra in *R*-space for as-prepared and activated Ag_25_/carbon catalysts and Ag foil reference.

XAS experiments were carried out to further examine the structural integrity of Ag_25_ clusters on carbon supports. While XANES spectroscopy gives information about the oxidation state and local environment, EXAFS spectroscopy affords information about the average coordination number (CN) around metal atoms and bond distances.^57^Fig. 5 shows the Ag K-edge spectra obtained for as-prepared and activated Ag_25_/carbon catalysts, alongside that of Ag foil as a reference. The spectra show a strong K-edge feature for Ag at 25 514 eV which is attributed to dipole-allowed 1s to 5p transition.^58^Fig. 5(a) shows the XANES spectra of the sample; the peak at *ca.* 25 550 eV is absent in the as-prepared clusters and grows in intensity with increased activation temperature. A number of groups, including our own, have observed that this multiple scattering feature is a good fingerprint for Au cluster growth in analogous Au cluster samples (*i.e.* small clusters do not have such multiple scattering peaks given their small size).^12,17,18^ This agrees well with previous TEM results from these samples. The as-prepared sample shows distinct near edge features to suggest that the neighboring atoms are different for the as-prepared sample as compared to activated samples. Comparing the near edge features with those of reference Ag foil, it shows that Ag–Ag interactions become more predominant at calcination temperatures above 250 °C which affirms that cluster sintering becomes problematic beyond 250 °C. This agrees with the results from both TGA and XPS, confirming the removal of protecting thiolate ligands upon thermal activation of as-prepared Ag_25_/carbon catalyst. It is noteworthy that the spectra features of all the samples show no detectable Ag_2_O or Ag_2_S in these samples, in consistent with the XPS results (Fig. 3, 4 and S1, ESI†).

Fig. 5(b) shows the Ag K edge EXAFS spectra of the Ag_25_/carbon samples in *R*-space. EXAFS measurements are sensitive to the bonding environment of the absorbing atom. All samples, as well as the reference Ag foil, show major peaks at around 2.72 Å (note the plotted data has no phase shift correction) that are characteristic features of first shell Ag–Ag contributions (Fig. 5(b)), while only the as-prepared sample has a Ag–S feature in the 1.85 Å region. The crystallographic information of Ag_25_L_18_^−^ provided in the literature was used to make the theoretical model for fitting the experimental data obtained for as-prepared sample using a two-shell fitting approach.^39^ A similar approach was reported for EXAFS fittings of Ag_44_(SR)_30_^4−^ clusters.^20^ The fitting parameters such as CN, nearest neighbour distance (*R*), Debye–Waller factors (*σ*^2^), and energy shift (*E*_o_) are presented in Table S2 (ESI†), while the k-space and R-space plots are shown in Fig. S2 (ESI†). The bond distance of 2.401(6) Å was obtained for the Ag–S contribution from the fit and this value agrees very well with values of 2.382–2.445 Å earlier reported for Ag–S bond in the crystal structure,^39^ and a value of 2.81(2) Å was obtained for Ag–Ag bond distance in the as-prepared sample. CN values of 1.08(5) and 0.5(1) were obtained for Ag–S and Ag–Ag bonds, respectively. We note that the CN value for first shell Ag–Ag interactions is low but this can be due to both the presence of multiple first shell Ag–Ag contributions and perhaps low levels of Ag thiolate impurities. Attempts to use multiple Ag–Ag first shells in fitting were unsuccessful as the data quality was not sufficient for such a detailed analysis.^18,59^ Nevertheless, the data suggests successful immobilization of Ag_25_L_18_^−^ clusters onto carbon supports without compromising their structural integrity.^39^ In summary, XPS and XAS data of as-prepared Ag_25_/carbon catalysts suggest that the structural changes in activated Ag_25_/carbon samples can be solely due to calcination which causes removal of protecting ligands from Ag_25_L_18_^−^ clusters.

In addition to the observable changes in the near edge features in XANES spectra (Fig. 5(a)), a single-shell fit approach is employed to analyze the EXAFS data obtained for thermally treated Ag_25_/carbon samples to probe the growth in cluster size with increasing activation temperatures. The single-shell fit entails the use of a Ag fcc model and is adopted due to observed growth in cluster size and presence of zerovalent Ag as revealed by the other results. Fig. S3 (ESI†) shows both *k*-space and *R*-space EXAFS spectra at the Ag K-edge for the thermally activated Ag_25_/carbon samples, and the obtained fitting parameters are presented in Table 1. The first shell Ag–Ag coordination number (CN) increases with an increase in activation temperature; this agrees with the increase in the peak intensities of the *R*-space EXAFS spectra (Fig. 5(b)). No significant Ag–S contributions were found for any of the calcined samples. For samples thermally activated at 250 °C, 350 °C, and 450 °C, the first shell Ag–Ag CN values are 3.8(7), 6.1(3), and 6.3(3), respectively. A CN value of 3.8(7) for sample activated at 250 °C and 6.1(3) for sample activated at 350 °C suggest significant structural changes occur at higher calcination temperatures due to sintering of Ag clusters. There is no significant change in the Debye–Waller parameter which indicates that the degree of disorder remains unchanged with the increase in activation temperature. However, the Ag–Ag bond distance slightly increases with activation temperature which is also consistent with an increase in cluster size.^20,58,60^ Altogether, it can be said that the protecting thiolate ligands are completely removed with little compromise in the size of Ag_25_ clusters by thermal activation at 250 °C, whereas higher temperature calcination leads to moderate cluster sintering.

**Table 1:** Single shell EXAFS fitting parameters for activated Ag_25_/carbon catalysts

Catalyst	CN (Ag–Ag)	*R* Å^−1^ (Ag–Ag)	*σ* ^2^/Å^2^ (Ag–Ag)	*E*_o_ shift/eV (Ag–Ag)	*R*-factor
@250 °C	3.8(7)	2.826(6)	0.0080(2)	−3.7(8)	0.017
@350 °C	6.1(3)	2.854(5)	0.0090(7)	−1.9(5)	0.019
@450 °C	6.3(3)	2.859(6)	0.0081(8)	−0.7(6)	0.019

Estimation of average particle sizes (*d*_mean_) directly from EXAFS CNs is reported in the literature.^61–63^ Recently, Wei *et al.* (2013) reported an equation that quantitatively relates the CN from EXAFS fittings to average particle size using Ag nanoparticle sizes obtained by analyzing STEM images (for particles ranging from 0.8 to 8.7 nm in size).^63^ The equation was modified to give eqn (1) below that can directly estimate the average particle size in nanometers (nm). Using the concept of error propagation,^64^eqn (2) was derived to estimate the error in *d*_mean_ in eqn (1). These two equations enable estimation of size distribution using CN errors from EXAFS fittings.

1 *d*_mean_ (nm) = 10^((0.1319 × CN)−0.5763)^

2 *σ*_d_ = |*d*(0.1319 × ln(10)*σ*_CN_)|

Using the two equations above, the average cluster size was estimated to be 0.84 ± 0.18 nm, 1.69 ± 0.15 nm, and 1.80 ± 0.16 nm for Ag_25_/carbon samples activated at 250 °C, 350 °C and 450 °C respectively. These values are somewhat consistent with the average cluster sizes measured from TEM images of 1.2 ± 0.3 nm, 2.6 ± 0.4 nm, and 2.8 ± 0.6 nm, respectively (Fig. 2), though TEM measurements slightly over-represent cluster sizes. Determining precise particle sizes and distributions when dealing with clusters in the nanometer size range by TEM can be difficult in that sub-nanometer clusters may be present that are not easily observed. Thus, as the TEM particle sizes for the three activated samples are larger than the mean diameters calculated from first-shell CN values, this suggests there may be a population of smaller clusters which are difficult to image by TEM in the samples.

Selective oxidation of olefins to epoxides is a desirable chemical conversion in the fine chemical industry, and the use of heterogeneous Ag-based catalysts has gained attention in recent years.^65^ Specifically, styrene to styrene oxide (SO) conversion is a desirable chemical process in industry as SO is a useful chemical in the production of surface coatings and cosmetics.^65,66^ SO is also beneficial to the production of styrene glycol, polyurethanes, and cross-linked polyesters.^66^ Both activity and selectivity are important parameters for describing performance of catalysts in a given reaction. Table 2 presents the results obtained for the catalytic evaluation of Ag_25_/carbon as catalysts in styrene oxidation reactions. Catalytic activity was measured in terms of both conversion and an average turnover number (TON), which was determined as moles of converted styrene per moles of Ag in the catalyst. No activity was observed in blank reactions (without catalyst or carbon support) and about 1% conversion of styrene was recorded when air was used as oxidant instead of *tert*-butyl hydroperoxide (TBHP), in the presence of our catalysts. Significant activity was observed with TBHP as an oxidant and thus data obtained for styrene oxidation reaction with TBHP were used to compare the performances of studied catalysts. Interestingly, carbon supports alone showed about 15% conversion of styrene, on average, under our reaction conditions. The results show that Ag_25_/carbon catalysts display size-dependent activities with SO as a major product for all examined catalysts in this study. The range of values obtained for selectivity for styrene oxide is in agreement with those reported for Au_25_ clusters in literature.^10,13^ The TON of as-prepared catalysts was found to be 1114 and it increased to 1325 for catalysts activated at 250 °C. This increase in TON value can be ascribed to significant removal of protecting ligands off the catalyst surface which enables better access of the active sites by the substrate. The subsequent drop in TON values for catalysts activated at higher temperatures is due to particle sintering as indicated by the increase in the CN values in the XAS measurements and average particle sizes in the TEM measurements. The small trend obtained for product selectivity may be due to particle size effects, as the clusters increase in size at higher calcination temperatures.

**Table 2:** Catalytic performances of support, as-prepared and activated Ag_25_/carbon catalysts^a^

Catalyst	Activity		Selectivity	
	Conversion (%)	TON	SO (%)	BA (%)
Carbon support	15.3		90.9	9.1
Ag_25_/carbon as-prepared	51.6	1114	95.1	4.9
Ag_25_/carbon@250 °C	61.4	1325	94.0	6.0
Ag_25_/carbon@350 °C	49.9	1078	92.6	7.4
Ag_25_/carbon@450 °C	46.6	1006	89.4	10.6

a Reaction conditions: styrene (8.0 mmol), TBHP (24 mmol), MeCN (5.0 mL), catalyst (20 mg) in a flask under reflux, stirring speed (600 rpm), reaction temperature of (82 °C), reaction time (24 h). Ratio of substrate to metal is 2157 : 1. TON = moles of product/moles of Ag.

Others have shown that TBHP works as a radical initiator, when present in sub-stoichiometric quantities, which then allows the reaction to proceed with oxygen as the main oxidant.^67,68^ Using the Ag_25_/carbon@250 °C catalyst, we performed reactions with sub-stoichiometric amounts of TBHP and for longer reaction times, and the results are presented in Table 3. The reduction in amount of TBHP from 24 mmol (*i.e.* 300% of styrene amount) to 0.40 mmol (*i.e.* 5% of styrene amount) leads to a decrease in the styrene conversion from 61.4% to 5.8% for a reaction period of 24 h, and no conversion is seen in the complete absence of TBHP initiator. However, by extending the reaction period to 72 h, a conversion of 23.3% is observed with 0.40 mmol TBHP. This indicates that the reaction proceeds at a much slower rate when catalytic amounts of TBHP are used as radical initiator and molecular oxygen is the major oxygen atom source for the epoxide formation. Similar observations were reported in separate studies for Au and Ag nanoparticle catalysts.^68,69^ Thus, a radical mechanism is occurring for styrene epoxidation over the activated Ag_25_/carbon catalysts, in which the thermolysis of *t*-BuOOH (TBHP) occurs on the surface of silver particles to give *t*-BuOO˙ and H˙ radicals, as reported for similar systems.^68,70^ It is of note that the selectivity towards epoxide formation is tremendously reduced when using sub-stoichiometric amounts of TBHP. The decrease in the selectivity is consistent with results in the literature as TBHP as an oxidant has been identified to be more selective for epoxide formation.^69^ The spent catalyst was subjected to a recycling process that involves washing the spent catalyst with acetone for three times, followed by oven drying at 110 °C for 30 min. Thereafter, the recycled catalyst was used for two more cycles of reaction and it was found to have slightly higher activity and similar selectivity. The increase in activity after the first cycle of reaction may be due to the removal of organic sulfur species adsorbed on the catalyst surface by washing steps utilized to recycle the catalyst.

**Table 3:** Catalytic performance of activated Ag_25_/carbon@250 °C catalyst with sub-stoichiometric amounts of TBHP^a^

Entry		Activity		Selectivity	
Cycle	Reaction time	Conversion (%)	TON	SO (%)	BA (%)
First	24 h	5.8	125	60.8	39.2
	72 h	23.3	502	59.6	40.4
Second	24 h	7.2	155	59.8	40.2
	72 h	46.2	997	61.0	39.0
Third	24 h	7.4	160	59.6	40.4
	72 h	46.7	1007	61.2	38.8

a Reaction conditions: styrene (8.0 mmol), TBHP (0.4 mmol), MeCN (5.0 mL), catalyst (20 mg) in a flask under reflux, stirring speed (600 rpm), reaction temperature of (82 °C). Ratio of substrate to metal is 2157 : 1. TON = moles of product/moles of Ag.

## Conclusions

Atomically precise Ag_25_L_18_^−^ clusters were synthesized and deposited onto carbon supports for catalysis. Carbon-supported Ag_25_ clusters were activated at mild temperatures (250 °C) without sintering, while growth in particle sizes was observed at higher calcination temperatures. Upon thermal activation, the protecting thiolate ligands were detached from the surface of Ag_25_ clusters and the products of dethiolation were adsorbed onto the carbon supports, and these sulfur species were further removed at high temperatures. XPS and XAS data both showed that the resulting activated Ag clusters have metallic character, and Ag_2_S formation was not observed. Both as-prepared and activated Ag_25_/carbon catalysts showed high selectivity towards styrene oxide, with maximum activities and selectivities seen for clusters activated at 250 °C. The catalytic performance of the catalyst was dramatically reduced when sub-stoichiometric, catalytic amounts of TBHP were used. Results showed that the reaction progressed much more slowly and with much lower selectivity using O_2_ as the major oxygen source for epoxide formation, which suggests that TBHP is a better oxidant for the highly selective formation of epoxides. Future work will focus on using such activated Ag_25_/carbon catalysts as starting materials for the design of Ag based bimetallic clusters with other metals using galvanic replacement methods.

## Conflicts of interest

There are no conflicts to declare.

## Supplementary Material

RA-009-C9RA05566E-s001
