# Supplementary material for: Activation of atomically precise silver clusters on carbon supports for styrene oxidation reactions
Source: RSC Adv. 2019 Sep 6;9(48):28019–27. doi: 10.1039/c9ra05566e (PMC9070844; doi:10.1039/c9ra05566e)
Supplement: RA-009-C9RA05566E-s001 [file RA-009-C9RA05566E-s001.pdf]

Electronic Supplementary Material (ESI) for RSC Advances.
This journal is © The Royal Society of Chemistry 2019

Supplementary Information

Activation of Atomically Precise Silver Clusters on Carbon Supports for Styrene Oxidation Reactions

Kazeem O. Sulaiman, V. Sudeeshkumar, and Robert W. J. Scott*

Department of Chemistry, 110 Science Place, University of Saskatchewan, Saskatoon, Saskatchewan S7N 5C9, Canada

*robert.scott@usask.ca

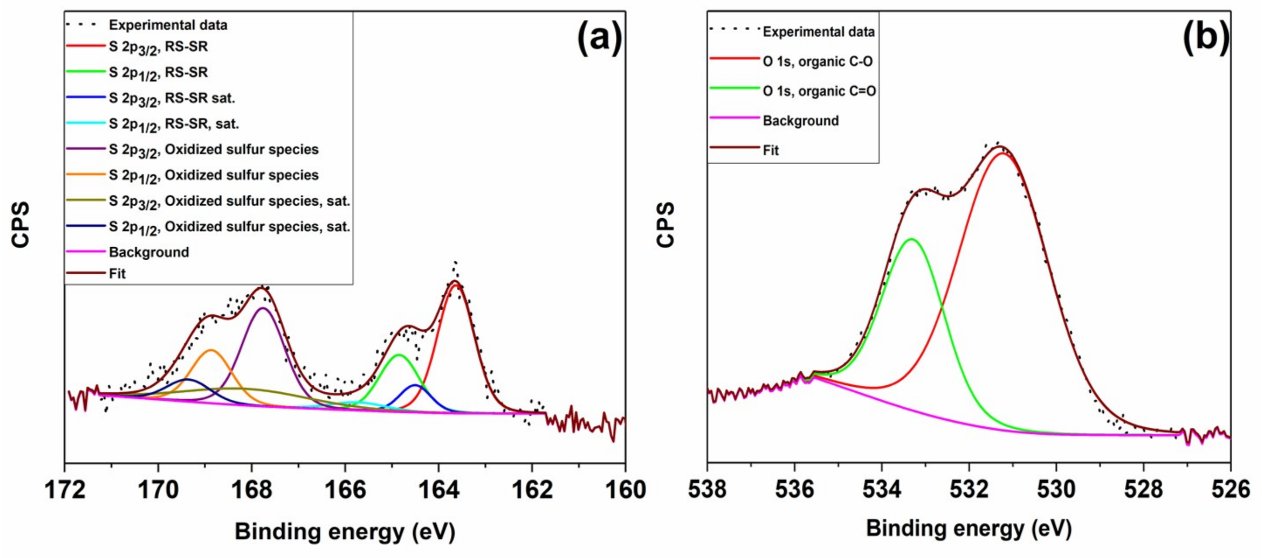

Fig. S1. XPS spectra for activated Ag_{25}/carbon catalyst at 350 °C; (a) S 2p, and (b) O 1s spectra.

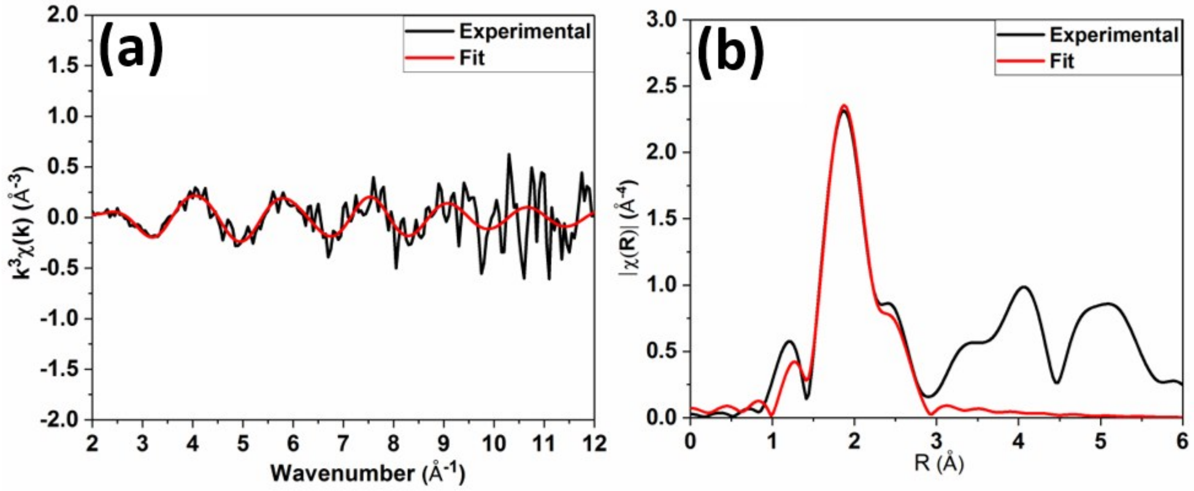

Fig. S2. EXAFS data in (a) k-space and (b) R-space, for as-prepared Ag_{25}/carbon catalyst.

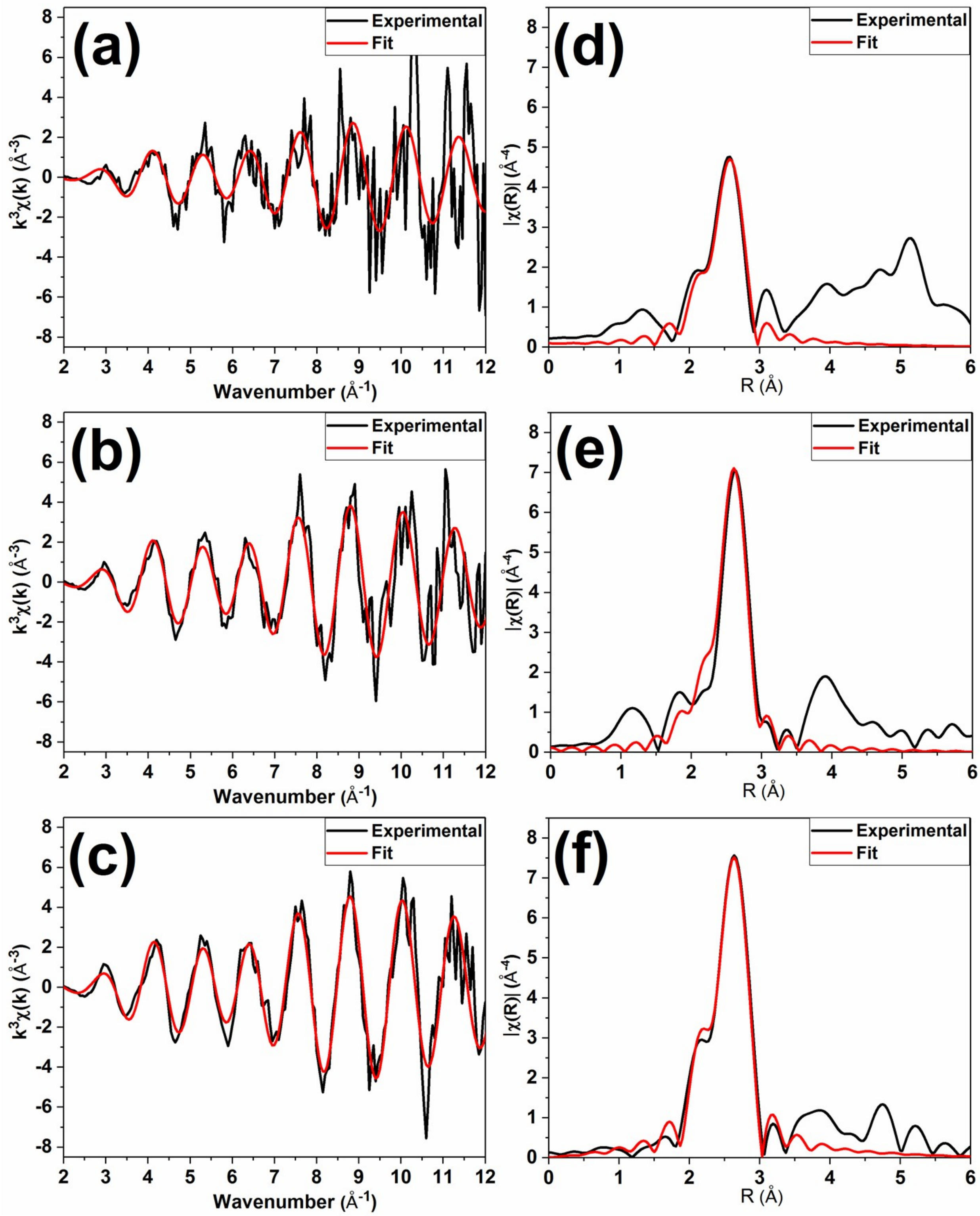

Fig. S3. EXAFS data in k-space (a-c) and R-space (d-f) for activated Ag_{25}/carbon catalysts at 250 °C (a,d), 350 °C (b,e), and 450 °C (c,f) respectively.

Table S1. Stability of dispersed $Ag_{25}L_{18}^{-}$ clusters as determined by the preservation of optical transitions in UV-Vis measurements.

**Solvent property**		**Longevity (in days) at different storage temperature**	
**Solvent**	**Dielectric constant (ε)**	**22-24 °C (ambient)**	**~4 °C (cold)**
THF	7.58	2	24
DCM	8.93	4	31
DMF	36.7	6	120
DMSO	46.7	10	2*

*Freezes up after 2 days.

Table S2. Multi-shell EXAFS fitting parameters for as-prepared Ag_{25}/carbon catalysts.

**Bond type**	**CN**	**R/Å**	**σ^2/Å^2**	**E_o shift /eV**	**R-factor**
Ag-S	1.08(5)	2.401(6)	0.00272(2)	-4.0(8)	0.0066
Ag-Ag	0.5(1)	2.81(2)	0.00900(3)	-4.0(8)	
